# The Contributory Role of Cell Blocks in Salivary Gland Neoplasms Fine Needle Aspirations Classified by the Milan System for Reporting Salivary Gland Cytology

**DOI:** 10.3390/diagnostics11101778

**Published:** 2021-09-27

**Authors:** Erkka Tommola, David Kalfert, Heli Hakso-Mäkinen, Ivana Kholová

**Affiliations:** 1 Fimlab Laboratories, Department of Pathology, Tampere University Hospital, 33520 Tampere, Finland; erkka.tommola@tuni.fi (E.T.); heli.hakso-makinen@fimlab.fi (H.H.-M.); 2 Faculty of Medicine and Health Technology, Tampere University, 33520 Tampere, Finland; 3 Department of Otorhinolaryngology and Head and Neck Surgery, First Faculty of Medicine, University Hospital Motol, Charles University, 15006 Prague, Czech Republic; david.kalfert@fnmotol.cz

**Keywords:** salivary glands, FNA, cell block, The Milan System for Reporting Salivary Gland Cytopathology

## Abstract

(1) Background: The Milan System for Reporting Salivary Gland Cytopathology (MSRSGC) was introduced in 2018, bringing an organ-specific classification system for salivary gland cytopathology. The aim of present study is to evaluate the MSRSGC prospectively, based on a two-year experience in the tertiary care center pathology department, and evaluate the role of routine cell block (CB) preparation in salivary gland cytopathological diagnostics. (2) Methods: In our institution, the Department of Pathology, Fimlab Laboratories, Tampere, Finland, the MSRSGC has been implemented in salivary gland cytopathology since January 2018 and, over a two-year period (January 2018–December 2019), there were 365 fine-needle aspirations, of which 164 had a surgical follow-up. The CB methods used were Plasma-thrombin, the collection of visible fragments, and the Shandon and in-house methods. (3) Results: The MSRSGC diagnostic figures were as follows: accuracy 87.5%, sensitivity 45.8% and specificity 98.9%. For diagnostic categories of MSRSGC (non-neoplastic, benign neoplasm and malignant neoplasm) (*n* = 63) diagnostic accuracy was 98.4%, and for undetermined categories (atypia of undetermined significance, salivary gland neoplasm of uncertain malignant potential and suspicious for malignancy) (*n* = 49) diagnostic accuracy was 73.5%. Non-contributory cell blocks resulted more often in a false negative diagnosis (25%, 3/12) than a true negative diagnosis (10%, 7/73, *p* < 0.001), and is, most likely, an insufficient cytological diagnosis (86%, 18/21, *p* < 0.001). (4) Conclusion: The application of MSRSGC and CBs are beneficial in salivary gland cytological diagnosis, increasing diagnostic accuracy and, thus, patients’ management and treatment.

## 1. Introduction

The Milan System for Reporting Salivary Gland Cytopathology (MSRSGC) was introduced in 2018 following other organ specific cytopathological reporting. It aimed for better patient care, bringing a practical, evidence-based, user-friendly classification system with characterization and management algorithms [1,2]. Several international studies have stated that the MSRSGC is a reliable tool for salivary gland cytopathology categorization [3,4,5,6,7]. Cell blocks (CBs) are a collection of sediments and visible pieces of tissue from cytological specimens that are concentrated and processed into paraffin blocks, and stained with hematoxylin-eosinike surgical specimens. Various techniques can be used to prepare CBs, with each method having their advantages and disadvantages [8,9,10]. In our institution, cytological material is triaged, and one of the following CB methods is applied: the plasma-thrombin method, the collection of visible tissue fragments, the in-house method [8,11] or the Shandon method.

The aim of the present study was to evaluate the MSRSGC prospectively, based on a two-year experience in the tertiary care center pathology department, and evaluate the role of routine CB preparation and CB methods in salivary gland cytopathological diagnostics.

## 2. Materials and Methods

At the Department of Pathology, Fimlab Laboratories, Tampere, Finland, all salivary fine needle aspirations (FNAs) were given cytopathological diagnoses according to the MSRSGC since January 2018 [7]. CBs were routinely prepared in all cases. The study material consisted of all salivary gland fine needle aspirations during a two-year-period from January 2018 until December 2019, which were searched from an electronic pathology database. Cases with surgical follow-up were analyzed, and for each MSRSGC category the risk of neoplasm (RON) was calculated by all neoplastic cases over all cases with surgical follow-up; the risk of malignancy (ROM) was calculated by all malignant cases over all cases with surgical follow-up; and the overall risk of malignancy was calculated by all malignant cases over all cases with or without surgical follow-up. The diagnostic accuracy of the MSRSGC in the present study was determined for diagnostic categories (non-neoplastic, benign neoplasm and malignant neoplasm), undetermined categories (atypia of undetermined significance, salivary gland neoplasm of uncertain malignant potential and suspicious for malignancy), and all diagnostic and undetermined categories together. Closer attention was paid to each case with neoplastic surgical follow-up with the CB available (*n* = 111). In the cases with neoplastic histopathological diagnoses, the cytology was evaluated and the technique and role of the routinely prepared CB was assessed. FNAs were performed by radiologists with 22 G needles under ultrasound control. Samples were alcohol-fixed, cytospun and stained with Papanicolaou stain. CBs were prepared with the plasma-thrombin method in 33 (46.5%) cases, the collection of visible tissue fragments in 29 (40.8%) cases, the Shandon method in 6 (8.5%) cases and the in-house method in 3 (4.2%) cases. Plasma-thrombin CB was made by adding plasma and thrombin to cellular sediment that is allowed to clot [8]. When visible small tissue fragments were present, the CB specimens were treated as a small biopsy [8]. The Shandon method was performed according to manufacturer protocol (Thermo Fisher Scientific, Waltham, MA, USA). The in-house method was previously described in detail [11]. The role of CBs was divided into three categories: non-contributory, supporting diagnosis and crucial for diagnosis, based on the relevance and advantages CBs offered for cytological diagnosis. Categorization was carried out for this study as follows: non-contributory CBs were mainly acellular or of low cellularity with no impact on final diagnosis; CBs supporting the diagnosis usually contained the same or similar diagnostic elements as cytological preparation; and CBs crucial for diagnosis consisted of material that gave diagnostic information superior to the information revealed from cytological preparation.

Statistical analysis was performed for the correlation of the role of CBs with cellularity, the CB preparation method, immunocytochemistry (ICC), and MSRSGC categories, as well as the cyto-histological correlation of benign and malignant neoplasms. Histopathological diagnosis was held as the gold standard and cytological diagnosis was compared to determine the correlation regarding malignant neoplasm.

All statistical analyses were performed using IBM SPSS Statistics (version 22.0; SPSS, IBM, Armonk, NY, USA). Mean and range were counted. The statistical results were calculated using Fisher’s exact test (two-tailed) and the Pearson test; and *p* values equal or to less than 0.05 were considered as significant. Sensitivity, specificity, accuracy, positive predictive value (PPV) and negative predictive value (NPV) were calculated. The 95% confidence interval (95% CI) for sensitivity, specificity, accuracy, PPV and NPV were calculated.

## 3. Results

During a two-year period, 365 salivary gland FNAs were diagnosed according to the MSRSGC, and 164 (44.9%) of them had a surgical follow-up with histopathological diagnosis. The topographical localization was in the parotid gland in 139 (82%) cases and the submandibular gland in 25 (18%) cases. Detailed distribution according to MSRSGC categories can be found in Supplementary Table S1. In total, 86 (52.4%) of the cases were male and 78 (47.6%) were female. The average lesion size was 2.3 cm (0.8–5.0 cm), the average patient age was 58.3 (13–95) years and the clinical characteristics for each MSRSGC category are presented in Table 1.

**Figure 1:**
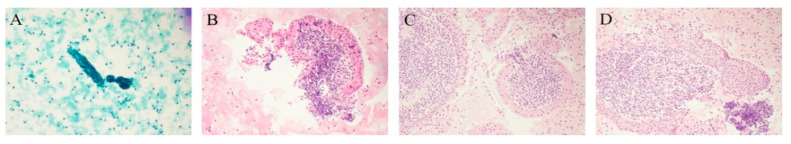
The most common benign tumor in our series, Warthin tumor, is often hypocellular cytologically (**A**), but CBs contain diagnostic papillae lined by oncocytic epitelium with lymphatic stroma (**B**,**C**) with occasional squamous cell metaplasia (**D**). Original magnification: 100× (**A**), 200× (**B**–**D**).

The risk of malignancy was 22.0% for all salivary gland lesions with surgical follow up. For each MSRSGC category, the risk of malignancy was as follows: non-diagnostic (*n* = 52) 23.1%, non-neoplastic (*n* = 4) 25.0%, atypia of undetermined significance (*n* = 25) 4.0%, benign neoplasm (*n* = 53) 0.0%, neoplasm of uncertain malignant potential (*n* = 27) 29.6%, suspicious for malignancy (*n* = 6) 83.3% and malignant neoplasm (*n* = 6) 100.0% (Table 1). The most common benign neoplasm was Warthin’s tumor (*n* = 49) (Figure 1), and the most common malignant neoplasm was carcinoma ex PA (*n* = 7), forming 36.3% and 5.2% of all salivary gland neoplasms, respectively. All tumors are listed according to MSRSGC categories in Supplementary Table S2.

The FNAs accuracy, sensitivity and specificity in differentiating between benign and malignant neoplasms (*n* = 112) were 87.5%, 45.8%, and 98.9%, respectively. For diagnostic categories (non-neoplastic, benign neoplasm and malignant neoplasm) (*n* = 63) the diagnostic accuracy was 98.4%, and for undetermined categories (atypia of undetermined significance, salivary gland neoplasm of uncertain malignant potential and suspicious for malignancy) (*n* = 49) the diagnostic accuracy was 73.5% (Table 2).

**Figure 2:**
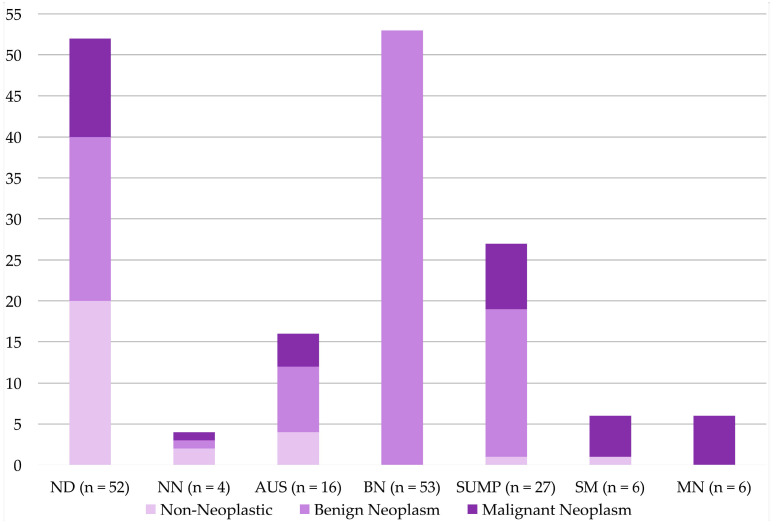
Milan System for Reporting Salivary Gland Cytopathology: histological diagnoses of non-neoplastic, benign neoplasm and malignant neoplasm in each Milan category.

Deviations of non-neoplastic, benign neoplastic and malignant neoplastic cases are illustrated in Figure 2.

The present study material consisted of 111 histologically proven neoplastic cases with the CBs available. CBs with less than 10 cells were all (*n* = 28) non-contributory for diagnosis, and those with 10–50 cells (*n* = 24) were crucial for diagnosis in 21%, supported the diagnosis in 75% and were non-contributory in 4% of cases (*p* < 0.001) (Table 3).

**Figure 3:**
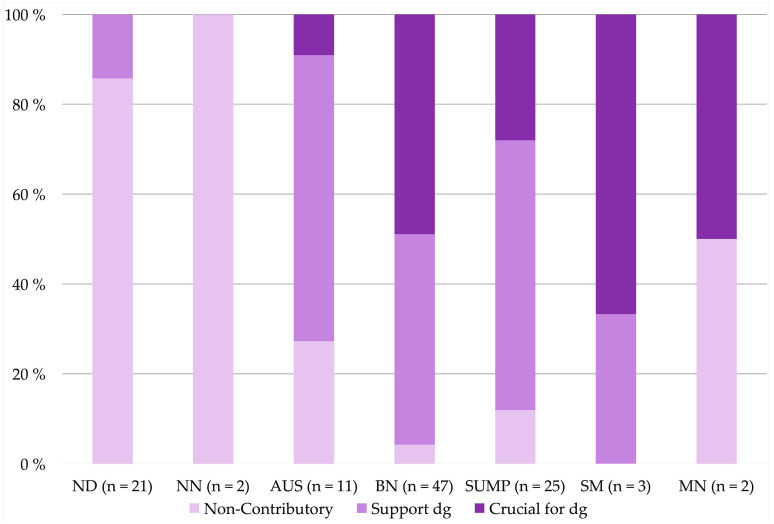
Milan System for Reporting Salivary Gland Cytopathology: cell block contributions of non-contributory, supporting diagnosis and crucial for diagnosis in each Milan category.

Out of the four methods used for the preparation of the CBs, the Shandon method was the most likely (4/9 cases) to be non-contributory (*p* = 0.047). Non-contributory CBs resulted most often in the MSRSGC insufficient category (18/21 of insufficient cases, *p* < 0.001) and the least in the benign neoplasm category (2/47 cases of benign neoplasm category, *p* < 0.001), as shown in Table 3 and illustrated in Figure 3.

**Figure 4:**
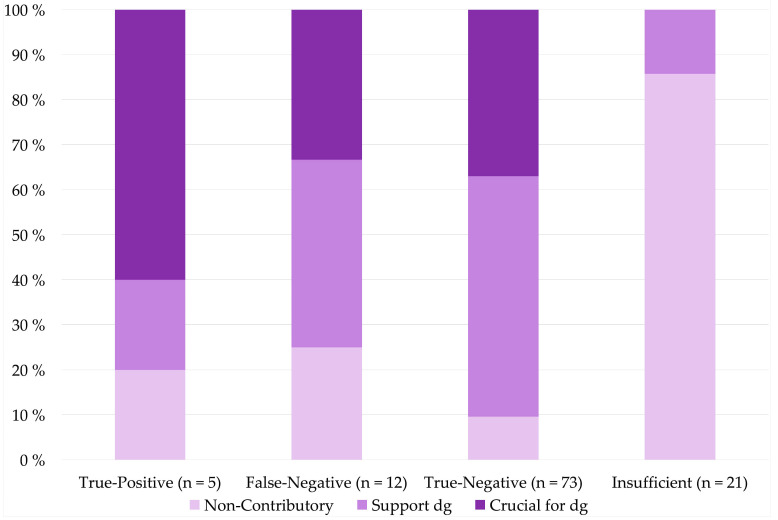
Cell block contribution by cyto-histological correlation.

CBs were crucial for or supported the diagnosis in 90% (66/73) of true-negative cases regarding malignant neoplasm (*p* < 0.001), in 80% (4/5) of true-positive cases and in 75% (9/12) of false-negative cases (Figure 4).

Thirteen false negative cases and one false positive case regarding malignancy were reported during the two-year study period. Only 1/14 of false cases were in the determined category. Extranodal marginal zone B-cell lymphoma of MALT type was diagnosed in the non-neoplastic category of the MSRSGC (Table 4). The CB, in this case, was non-contributory.

**Figure 5:**
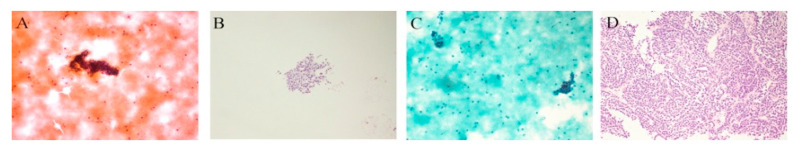
A cytologically-hypocellular case categorized as AUS (**A**,**B**) was, histologically, a basal cell adenoma. A SUMP-categorized cellular rich case was, histologically, a pleomorphic adenoma (**C**,**D**). Original magnification: 100× (**A**,**C**), 200× (**B**,**D**).

In the AUS category of MSRSGC there were four false-negative cases: Follicular lymphoma (*n* = 2), Adenoid cystic carcinoma (*n* = 1) and Myoepithelial carcinoma ex PA (*n* = 1). Cell blocks were contributory in three cases and supported the diagnosis in one case (Figure 5A,B).

The majority of the false-negative diagnoses (8/13) were in the SUMP category: Carcinoma ex pleomorphic adenoma (*n* = 3), Acinic cell carcinoma (*n* = 2), Adenoid cystic carcinoma (*n* = 2) and Myoepithelial carcinoma ex pleomorphic adenoma (*n* = 1). Interestingly, and to highlight the overlapping cytopathological features of salivary gland neoplasms, there were also (*n* = 9) pleomorphic adenomas in the SUMP category (Figure 5C,D). In false negative cases, CBs were crucial for diagnosis in four cases (all cases of Carcinoma ex pleomorphic adenoma and Myoepithelial carcinoma ex pleomorphic adenoma) and supported the diagnosis in four cases.

When taking CB performance into account, CBs were crucial for or supported diagnosis most commonly in true-negative (90%, 66/73) and true-positive (80%, 4/5) cases. CBs were less likely to be crucial for diagnosis or support diagnosis in false-negative (75%, 9/12) cases. Moreover, CBs were least likely to be crucial for diagnosis in AUS (1/11) and SUMP (7/25) categories, promoting their uncertain nature. In insufficient cases (*n* = 21), only three CBs supported diagnosis (only normal tissue) which draws a link between insufficient cytological diagnosis and non-contributory cell blocks (*p* < 0.001).

**Figure 6:**
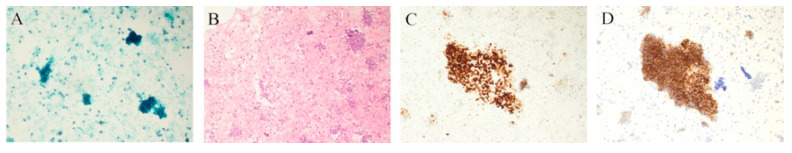
A case of small cell neuroendocrine carcinoma was categorized as malignant (**A**,**B**). Immunohistochemistry from CBs supported the diagnosis: INSM-1 positivity (**C**) and synaptophysin positivity (**D**). Original magnification: 100× (**A**), 200× (**B**–**D**).

In a separate analysis, the use of ICC in CBs was also analyzed, and its contribution to the final cytological diagnosis is summarized in Table 5. ICC was contributory in cellular CB samples (Figure 6) and the SUMP category with *p* ˂ 0.001.

## 4. Discussion

The cytopathological diagnosis of salivary gland lesions should take into account clinical features such as patient symptoms, physical examination and imaging, as well as lesion site and tumor frequencies [12]. Surgical management is decided in multidisciplinary meetings based on the above lesion characteristics in our hospital.

The MSRSGC, as a unified system, has defined diagnostic categories with predetermined ROMs and management recommendations. Furthermore, it enhances communication between pathologists and clinicians locally, nationally and internationally. [5,13].

During a two-year period of study, the overall accuracy of the MSRSGC in distinguishing between benign and malignant neoplasms in our institution was 87.5%. When only viewing the diagnostic categories of the MSRSGC (non-neoplastic, benign neoplasm and malignant neoplasm), accuracy was as high as 98.4%. In the three MSRSGC undetermined categories (AUS: atypia of undetermined significance, SUMP: neoplasm of uncertain malignant potential, suspicious for malignancy), the accuracy was 73.5%. Sensitivity in all categories was only 45.8%, but as high as 85.7% in diagnostic categories and as low as 29.4% in undetermined categories. Specificity was 98.9% in all categories, 100% in diagnostic categories and 96.9% in undetermined categories. The MSRSGC offers the undetermined categories between defined benign and malignant categories necessary for better management of cases with overlapping cytopathological features, due to heterogeneity of neoplastic lesions, neoplastic cell resemblance to normal salivary gland cells, metaplastic and/or cystic changes, and capsular invasion being impossible to evaluate in FNA material [13,14,15].

CBs are complimentary to conventional cytology, and are beneficial in several scenarios: (1) adding architecture dimensions to cytological material; (2) immunohistochemical characterization of cytological material; and (3) molecular techniques for further characterization of cytological material that may have a central role in targeted therapy in the future [8,16,17,18]. Nevertheless, CBs should not be a substitute for cytology [17].

CBs were reported as essential in head and neck cystic and metastatic lesions [19,20,21], but less data are available in salivary gland cytology [22], particularly in the salivary gland lesions classified by the MSRSGC [23,24]. In some MSRSGC institutional analyses, where CBs are routinely performed, their role in the diagnostic work-up was not evaluated [25,26]. In our present study, CBs were crucial for or supported the diagnosis in 90% of true-negative cases and in 80% of true-positive cases. Behaeghe et al. retrospectively analyzed 359 salivary gland samples processed only as a Cellient CB in view of the MSRSGC, with an overall accuracy of 92.9%, sensitivity of 75.9%, specificity of 97.9%, PPV of 91.7% and NPV of 95%, respective to the diagnostic categories (excluding non-diagnostic, AUS and SUMP categories) [23].

The limited cellularity of FNA specimens is a common limitation for CBs [10,27]. In our setting, radiologists in training are widely involved in FNAs and, due to the learning curve, there are high rates within the insufficient category. Pre-analytical issues, including triaging of cytological material, are crucial steps in the cytological diagnostic work-up [8,28,29]. In thyroid gland AUS/FLUS categorized specimens, cytological material triaging had a diagnostic input [28]. In the present study, CBs were contributory to (crucial for or supporting) the diagnosis in 72.7% of AUS cases, 95.7% of benign neoplasm cases, 88% of SUMP cases, 66.7% of suspicious for malignancy cases and 50% of malignant neoplasm cases, respectively. The CB contribution was statistically significant in the benign neoplasm category, where the CB was contributory to the diagnoses. On the other hand, in the non-diagnostic category, the non-contributory CB role was also statistically significant. Cellient CB processed in view of the MSRSGC, with a 33% non-diagnostic rate and 13.8% ROM in this category [23], supports the importance of triaging the cytological material to decrease non-diagnostic rates.

The need of ancillary techniques is increasing with the growing amount of known genetic mutations and rearrangements in salivary gland tumors [30]. This will further increase the diagnostic accuracy of salivary gland FNA and the MSRSGC. CBs are feasible as a material for ancillary molecular techniques [29]. In the European Federation of Cytology Societies survey, CBs were used for immunohistochemistry in 38% of laboratories, and 60% used a combination of material for immunohistochemistry [31]. Ancillary techniques were not deeply analyzed in the light of MSRSGC. In a Dubucs et al. study of 328 cases, immunohistochemistry contributed to a definitive diagnosis in 23.7% of cases and FISH in 33% of cases. Both techniques were applied on smears [32].

In practice, lymphoproliferative diseases are commonly misinterpreted in the FNA material of salivary glands. In our series, in addition to a false-positive case of extranodal marginal zone B-cell lymphoma of MALT type diagnosed in a non-neoplastic category, there were two false-negative follicular lymphomas in an AUS category. In all three cases, the CBs were non-contributory and contained less than 10 cells. In a series of 6249 reviewed cases performed by the College of American Pathologists, the lymphoma cases featured the highest false-negative rate at 57% [33]. In recent MSRSGC analyses, 10 lymphoma cases were misinterpreted in a non-neoplastic category and 9 lymphoma cases in an AUS category [4,23,25,26,34].

Another cytological pitfall is carcinoma ex pleomorphic adenoma (PA), which was the most common false-positive entity classified in undetermined categories in our series: one case of myoepithelial carcinoma ex PA was placed in a AUS category and another myoepithelial carcinoma ex PA in a SUMP category. In addition, the SUMP category contained three cases of carcinoma ex PA. The CBs in these cases were cellular, and either supported the neoplastic diagnosis or were crucial for the neoplastic diagnosis. To summarize other MSRSGC analyses, carcinoma ex PA cases were misinterpreted in a non-neoplastic category in three cases [6,23,35]: in an AUS category in one case [36] and in a SUMP category in only one case [4], but placement in a SUMP category is not generally rated as misinterpretation. Notably, nine cases were in a benign neoplasm IVA category [4,23,24,25]. On the other hand, 4 cytological diagnoses of carcinoma ex PA in a study by Pujani et al. were, histologically, a salivary duct carcinoma, squamous cell carcinoma, mucoepidermoid carcinoma and carcinoma NOS [36].

Interestingly, in one false-positive case of adenoid cystic carcinoma categorized as AUS, the CB was non-contributory, with cellularity less than 10 cells. In contrast, two cases each of adenoid cystic carcinoma and acinic cell carcinoma, categorized as SUMP, had cellular CBs which supported the neoplastic diagnoses in the present study. Of note, 43% of adenoid cystic carcinomas, acinic cell carcinomas and mucoepidermoid carcinomas were in a SUMP category in a large international multi-institutional study [37]. In an institutional study from the Memorial Sloan Kettering Cancer Centre, no acinic cell carcinoma nor adenoid cystic carcinoma were categorized as either a non-neoplastic or AUS category, but 33.3% of acinic cell carcinomas and 50% of adenoid were in a SUMP category [38].

## 5. Conclusions

The application of the MSRSGC and CBs are beneficial in salivary gland cytological diagnosis, increasing diagnostic accuracy and, thus, patients’ management and treatment. This study is limited by the short follow-ups of cases, as the MSRSGC was introduced in 2018 [1,7]. To increase communication with clinicians, false-positive result rates were recently recommended to be part of cytopathology reporting. In general, the false-positive rate is 10% in salivary gland FNAs [39].

## Figures and Tables

**Table 1:** MSRSGC: Clinical Characteristics and Risk of Malignancy.

MSRSGC Category	Patient Age Mean (Range)	Lesion Size Mean (Range)	No./Total No. (%)							
			Cases with Follow-Up		Risk of Neoplasm		Risk of Malignancy		Overall Risk of Malignancy	
Non-Diagnostic	62.6 (20–95)	2.1 (0.8–4.3)	52/139	(37.4)	32/52	(61.5)	12/52	(23.1)	12/139	(8.6)
Non-Neoplastic	36.3 (29–44)	1.9 (1.1–2.5)	4/16	(25.0)	2/4	(50.0)	1/4	(25.0)	1/16	(6.3)
AUS	53.6 (25–73)	2.5 (0.9–4.0)	16/45	(25.6)	12/16	(75.0)	1/25	(4.0)	1/45	(2.2)
Benign Neoplasm	54.0 (13–85)	2.6 (0.9–5.0)	53/105	(50.5)	53/53	(100)	0/53	(0.0)	0/53	(0.0)
SUMP	63.3 (24–88)	2.1 (1.3–5.2)	27/37	(73.0)	26/27	(96.3)	8/27	(29.6)	8/37	(21.6)
Suspicious for Malignancy	62.4 (48–79)	1.9 (1.0–3.0)	6/10	(60.0)	5/6	(83.3)	5/6	(83.3)	5/10	(50.0)
Malignant Neoplasm	67.0 (59–73)	2.1 (2.7–5.0)	6/14	(42.9)	6/6	(100)	6/6	(100)	6/14	(42.9)
Total	58.3 (13–95)	2.3 (0.8–5.0)	164/365	(44.9)	136/164	(82.9)	36/164	(22.0)	36/365	(9.9)

Abbreviations: MSRSGC, Milan System for Reporting Salivary Gland Cytopathology; AUS, Atypia of Undetermined Significance; SUMP, Neoplasm of Uncertain Malignant Potential.

**Table 2:** MSRSGC: Diagnostic Accuracy 2018–2019.

	*n*	Accuracy		Sensitivity		Specificity		PPV		NPV	
		(%)	95%CI (%)	(%)	95%CI (%)	(%)	95% CI (%)	(%)	95%CI (%)	(%)	95%CI (%)
All Categories	112	87.5	79.9–93.0	45.8	25.6–67.2	98.9	93.8–100.0	91.7	59.9–98.8	87.0	82.2–90.6
Diagnostic Categories ^1^	63	98.4	91.5–100.0	85.7	42.1–99.6	100.0	93.6–100.0	100.0	-	98.2	90.1–99.7
Undetermined Categories ^2^	49	73.5	58.9–85.1	29.4	10.3–56.0	96.9	83.8–99.9	83.3	38.8–97.5	72.1	65.4–77.9

^1^ (non-neoplastic, benign neoplasm, malignant neoplasm); ^2^ (atypia of undetermined significance, neoplasm of uncertain malignant potential, suspicious for malignancy).

**Table 3:** Contribution of Cell Blocks for Cytological Diagnosis was analyzed at four different levels: CB cellularity, CB method, MSRSGC categories and accuracy, which were compared with the CBs’ contributory role in the final cytological diagnosis.

Group for Analysis			*n*	Cell Block Contribution for dg *n* (%)								*p* Value
				Non-Contributory		Support dg		Crucial for dg		Contributory		
1	Cellularity	<10	28	28	(100%)	0	(0%)	0	(0%)	0	(0%)	**<0.001**
		10–50	24	1	(4%)	18	(75%)	5	(21%)	23	(96%)	
		>50	59	0	(0%)	30	(51%)	29	(49%)	59	(100%)	
2	Cell Block Method	Plasma-thrombin	53	14	(26%)	25	(47%)	14	(26%)	39	(74%)	1.000
		Visible Fragments ^1^	42	8	(19%)	18	(43%)	16	(38%)	34	(81%)	0.263
		Shandon	9	5	(56%)	4	(44%)	0	(0%)	4	(44%)	**0.047**
		In-house method	5	1	(20%)	1	(20%)	3	(60%)	4	(80%)	1.000
3	MSRSGC Categories	Non-Diagnostic	21	18	(86%)	3	(14%)	0	(0%)	3	(14%)	**<0.001**
		Non-Neoplastic	2	2	(100%)	0	(0%)	0	(0%)	0	(0%)	0.067
		AUS	11	3	(27%)	7	(64%)	1	(9%)	8	(73%)	1.000
		Benign Neoplasm	47	2	(4%)	22	(47%)	23	(49%)	45	(96%)	**<0.001**
		SUMP	25	3	(12%)	15	(60%)	7	(28%)	22	(88%)	0.076
		Suspicious for Malignancy	3	0	(0%)	1	(33%)	2	(67%)	3	(100%)	0.566
		Malignant Neoplasm	2	1	(50%)	0	(0%)	1	(50%)	1	(50%)	0
4	Accuracy	True-Positive	5	1	(20%)	1	(20%)	3	(60%)	4	(80%)	1.000
		False-Negative	12	3	(25%)	5	(42%)	4	(33%)	9	(75%)	1.000
		True-Negative	73	7	(10%)	39	(53%)	27	(37%)	66	(90%)	**<0.001**
		Insufficient	21	18	(86%)	3	(14%)	0	(0%)	3	(14%)	**<0.001**

^1^ Collection of Visible Tissue Fragments; Abbreviations: MSRSGC, Milan System for Reporting Salivary Gland Cytopathology. *p* values between non-contributory and contributory.

**Table 4:** Accuracy of Cytology Determining Malignancy.

		*n*	Cytology Determining Malignancy *n* (%)									
			True-Positive		False Positive		True-Negative		False-Negative		Insufficient	
Cell Block Method	Plasma-thrombin	53	5	(9%)	N.D.		30	(57%)	8	(15%)	10	(19%)
	Visible Fragments ^1^	42	0	(0%)	N.D.		32	(76%)	3	(7%)	7	(17%)
	Shandon	9	0	(0%)	N.D.		6	(67%)	1	(11%)	2	(22%)
	In-house method	5	0	(0%)	N.D.		4	(80%)	0	(0%)	1	(20%)
MSRSGC Categories	Non-Diagnostic	52	N.D.		N.D.		N.D.		N.D.		52	(100%)
	Non-Neoplastic	4	N.D.		N.D.		3	(75%)	1	(25%)	N.D.	
	AUS	16	N.D.		N.D.		12	(75%)	4	(25%)	N.D.	
	Benign Neoplasm	53	N.D.		N.D.		53	(100%)	0	(0%)	N.D.	
	SUMP	27	N.D.		N.D.		19	(70%)	8	(30%)	N.D.	
	Suspicious for Malignancy	6	5	(83%)	1	(17%)	N.D.		N.D.		N.D.	
	Malignant Neoplasm	6	6	(100%)	0	(0%)	N.D.		N.D.		N.D.	

^1^ Collection of Visible Tissue Fragments; Abbreviations: MSRSGC, Milan System for Reporting Salivary Gland Cytopathology; N.D., not determined.

**Table 5:** Contribution of ICC for Cytological Diagnosis was analyzed at four different levels: CB cellularity, CB method, MSRSGC categories and accuracy, which were compared with the CBs’ contributory role in final cytological diagnosis.

Group for Analyses			*n*	ICC Contribution for dg *n* (%)							*p*-Value
				No ICC		Supporting dg		Crucial for dg		Contributory	
1	Cellularity	<10	28	28	(100%)	0	(0%)	0	(0%)	0 (0%)	
		10–50	24	19	(79%)	4	(17%)	1	(4%)	5 (21%)	**<0.001**
		>50	59	33	(56%)	23	(39%)	3	(5%)	26 (44%)	
2	Cell Block Method	Plasma-thrombin	53	36	(68%)	13	(25%)	4	(8%)	17 (33%)	0.525
		Visible Fragments ^1^	42	29	(69%)	13	(31%)	0	(0%)	13 (31%)	0.668
		Shandon	9	8	(89%)	1	(11%)	0	(0%)	1 (11%)	0.285
		In-house method	5	5	(100%)	0	(0%)	0	(0%)	0 (0%)	0.319
3	MSRSGC Categories	Non-Diagnostic	21	21	(100%)	0	(0%)	0	(0%)	0 (0%)	**0.002**
		Non-Neoplastic	2	2	(100%)	0	(0%)	0	(0%)	0 (0%)	1.000
		AUS	11	9	(82%)	2	(18%)	0	(0%)	2 (18%)	0.514
		Benign Neoplasm	47	41	(87%)	5	(11%)	1	(2%)	6 (13%)	**0.003**
		SUMP	25	5	(20%)	20	(80%)	0	(0%)	20 (80%)	**<0.001**
		Suspicious for Malignancy	3	1	(33%)	0	(0%)	2	(67%)	2 (67%)	0.065
		Malignant Neoplasm	2	1	(50%)	0	(0%)	1	(50%)	1 (50%)	1.000
4	Accuracy	True-Positive	5	2	(40%)	0	(0%)	3	(60%)	3 (60%)	0.132
		False-Negative	12	4	(33%)	8	(67%)	0	(0%)	8 (67%)	**0.004**
		True-Negative	73	53	(73%)	19	(26%)	1	(1%)	20 (27%)	1.000
		Insufficient	21	21	(100%)	0	(0%)	0	(0%)	0 (0%)	**0.002**

^1^ Collection of Visible Tissue Fragments; Abbreviations: MSRSGC, Milan System for Reporting Salivary Gland Cytopathology. *p* values between no ICC and contributory.

## Data Availability

All data generated or analysed during this study are included in this article and its Supplementary Material files. Further enquiries can be directed to the corresponding author (I.K.).

## Supplementary Materials

The following are available online at https://www.mdpi.com/article/10.3390/diagnostics11101778/s1, Table S1: Topography of Salivary Gland Fine-Needle Aspirations, Table S2: MSRSGC: Histopathology Diagnoses of Neoplasms.
