# Supplementary material for: The Contributory Role of Cell Blocks in Salivary Gland Neoplasms Fine Needle Aspirations Classified by the Milan System for Reporting Salivary Gland Cytology
Source: Diagnostics (Basel). 2021 Sep 27;11(10):1778. doi: 10.3390/diagnostics11101778 (PMC8534546; doi:10.3390/diagnostics11101778)
Supplement: Supplementary file 1 [file diagnostics-11-01778-s001.zip › diagnostics-1346041-supplementary.pdf]

Supplementary Materials

The Contributory Role of Cell Blocks in Salivary Gland Neoplasms Fine Needle Aspirations Classified by Milan System for Reporting Salivary Gland Cytology

Erkka Tommola [1,2], David Kalfert [3], Heli Hakso-Mäkinen [1] and Ivana Kholová [1,2,*]

Table S1. Topography of Salivary Gland Fine-Needle Aspirations.

	Localization	
**MSRSGC Category**	**Parotid Gland**	**Submandibular Gland**
Non-Diagnostic	42 (76.2)	10 (23.8)
Non-Neoplastic	3 (66.7)	1 (33.3)
AUS	15 (93.3)	1 (6.7)
Benign Neoplasm	48 (89.6)	5 (10.4)
SUMP	21 (71.4)	6 (28.6)
Suspicious for Malignancy	5 (80.0)	1 (20.0)
Malignant Neoplasm	5 (80.0)	1 (20.0)
Total	139 (82.0)	25 (18.0)

Abbreviations: AUS, Atypia of Undetermined Significance; SUMP, Neoplasm of Uncertain Malignant Potential.

Table S2. MSRSGC: Histopathology Diagnoses of Neoplasms.

MSRSGC Category	Histopathology Diagnosis of Neoplasms	
	**Benign Neoplasm**	**Malignant Neoplasm**
Non-Diagnostic	Warthin's tumor (n = 13)<br>Cystadenoma (n = 4)<br>Basal cell adenoma benign (n = 1)<br>Lipoma (n = 1)<br>Pleomorphic adenoma (n = 1)	Extranodal marginal zone B-cell lymphoma, MALT-type (n = 3)<br>Adenoid cystic carcinoma (n = 2)<br>Basal cell adenocarcinoma (n = 2)<br>Burkitt lymphoma (n = 2)<br>Ductal carcinoma (n = 1)<br>Myoepithelial carcinoma ex PA (n = 1)<br>Squamous cell carcinoma, metastatic site (n = 1)
Non-Neoplastic	Warthin's tumor (n = 1)	Extranodal marginal zone B-cell lymphoma of MALT type (n = 1)
AUS	Warthin's tumor (n = 5)<br>Myoepithelioma (n = 1)<br>Basal cell adenoma benign (n = 1)<br>Pleomorphic adenoma (n = 1)	Follicular lymphoma (n = 2)<br>Adenoid cystic carcinoma (n = 1)<br>Myoepithelial carcinoma ex PA (n = 1)
Benign Neoplasm	Warthin's tumor (n = 29)<br>Pleomorphic adenoma (n = 22)<br>Myoepithelioma (n = 1)<br>Oncocytoma (n = 1)	
SUMP	Pleomorphic adenoma (n = 9)<br>Basal cell adenoma benign (n = 3)<br>Oncocytoma (n = 2)<br>Eccrine spiradenoma (n = 1)<br>Myoepithelioma (n = 1)<br>Oncoytic cystadenoma (n = 1)<br>Warthin's tumor (n = 1)	Carcinoma ex pleomorphic adenoma (n = 3)<br>Acinic cell carcinoma (n = 2)<br>Adenoid cystic carcinoma (n = 2)<br>Myoepithelial carcinoma ex PA (n = 1)
Suspicious for Malignancy		Myoepithelial carcinoma (n = 2)<br>Leiomyosarcoma, metastatic site (n = 1)<br>Salivary duct carcinoma (n = 1)<br>Squamous cell carcinoma, metastatic site (n = 1)
Malignant Neoplasm		Carcinoma ex pleomorphic adenoma (n = 1)<br>Ductal carcinoma (n = 1)<br>High grade neuroendocrine carcinoma (n = 1)<br>Malignant melanoma, metastatic site (n = 1)<br>Squamous cell carcinoma, basaloid (n = 1)<br>Squamous cell carcinoma, metastatic site (n = 1)

Abbreviations: MSRSGC, Milan System for Reporting Salivary Gland Cytopathology; AUS, Atypia of Undetermined Significance; SUMP, Neoplasm of Uncertain Malignant Potential.
